# A Comprehensive Study of Photorefractive Properties in Poly(ethylene glycol) Dimethacrylate— Ionic Liquid Composites

**DOI:** 10.3390/ma10010009

**Published:** 2016-12-24

**Authors:** Mostafa A. Ellabban, Gašper Glavan, Jürgen Klepp, Martin Fally

**Affiliations:** 1 Physics Department, Faculty of Science, Tanta University, Tanta 31527, Egypt; mostafa.ellaban@science.tanta.edu.eg; 2 Physics Department, Faculty of Science, Taibah University, P.O. Box 30002, Al-Madina Al-Monaoura 42353, Saudi Arabia; mellabban@taibahu.edu.sa; 3 Faculty of Mathematics and Physics, University of Ljubljana, Jadranska 19, SI-1000 Ljubljana, Slovenia; gasper.glavan1@student.fwf.uni-lj.si; 4 Faculty of Physics, University of Vienna, Boltzmanngasse 5, A-1090 Wien, Austria; juergen.klepp@univie.ac.at

**Keywords:** holographic gratings, mixed phase and absorption gratings, photopolymer composites, poly(ethylene glycol)dimethacrylate, ionic liquids, diffraction theories

## Abstract

A detailed investigation of the recording, as well as the readout of transmission gratings in composites of poly(ethylene glycol) dimethacrylate (PEGDMA) and ionic liquids is presented. Gratings with a period of about 5.8 micrometers were recorded using a two-wave mixing technique with a coherent laser beam of a 355-nm wavelength. A series of samples with grating thicknesses $d_{0}=10\ldots 150$ micrometers, each for two different exposure times, was prepared. The recording kinetics, as well as the post-exposure properties of the gratings were monitored by diffracting a low intensity probe beam at a wavelength of 633 nm for Bragg incidence. To obtain a complete characterization, two-beam coupling experiments were conducted to clarify the type and the strength of the recorded gratings. Finally, the diffraction efficiency was measured as a function of the readout angle at different post-exposure times. We found that, depending on the parameters, different grating types (pure phase and/or mixed) are generated, and at elevated thicknesses, strong light-induced scattering develops. The measured angular dependence of the diffraction efficiency can be fitted using a five-wave coupling theory assuming an attenuation of the gratings along the thickness. For grating thicknesses larger than 85 microns, light-induced scattering becomes increasingly important. The latter is an obstacle for recording thicker holograms, as it destroys the recording interference pattern with increasing sample depth. The obtained results are valuable in particular when considering PEGDMA-ionic liquid composites in the synthesis of advanced polymer composites for applications, such as biomaterials, conductive polymers and holographic storage materials.

## 1. Introduction

The realization of gratings in the sub-micrometer range was and still is a domain of holographic recording in photosensitive materials. For a long time, the latter were basically either photochromic or photorefractive crystals [1] or photosensitive polymers [2], which reached quite a mature state of development. The former offer typically excellent optical properties, thermal and temporal stability and allow the read-write option, but have a relatively low light-induced refractive-index modulation in the order of ≤$10^{-4}$. In contrast, polymers offer much larger refractive-index modulations (up to $10^{-2}$ [3]), but are less controllable, e.g., with respect to shrinkage or thermal stability. Over the last two decades, the focus moved to polymer composite materials, such as polymer liquid crystal composites (H-PDLC [4,5,6,7,8,9], POLICRYPS [10,11,12]) or polymer nanoparticle composites [13,14,15,16,17,18,19,20,21,22,23]. Another recently introduced composite consists of ionic liquids embedded in a polymer matrix [24], the material investigated in this study. Such composites have in common that they allow one to combine and make use of the individual advantages of their constituents. They are attractive candidate materials for applications, such as actuators, electrically-switchable elements, in distributed feedback lasers or diffractive optical elements for light and neutrons.

Ionic liquids (ILs) are organic salts in the liquid state at room temperature or below. They consist entirely of ions and have unique properties, such as negligible vapor pressure or excellent thermal stability. They are nonflammable in the liquid state in a wide range of temperatures, low or non-toxic, of a high chemical stability, high ionic conductivity and able to dissolve numerous organic, inorganic and organometallic compounds [25]. In addition, ILs are environmentally-friendly solvents in addition to being excellent absorbents of ${\mathrm{CO}}_{2}$ when added to other specific solvents [26,27]. Therefore, they are considered to be essential for green chemistry synthesis and have attracted much attention as potential alternatives to conventional volatile organic-solvent systems. Moreover, ILs have been exploited in diverse applications, such as in lithium batteries [28], as electrochemical sensors and biosensors [29], for the preparation of inorganic oxide nanostructures and biopolymers [30]. Using ILs as solvents and catalysts for different types of polymerization processes has many advantages; see, e.g., [27,31]. Those can be exploited to produce clean polymers and to reduce costs because of the recyclability of IL-catalysts.

Lin et al. investigated the influence of different ILs based on dialkylimidazolium as additives to poly(ethylene glycol)dimethacrylate [24,32,33]. They reported a significant improvement in the sensitivity, resolution and diffraction efficiency (from 1% in the absence of IL and a polymer binder to 34%) of holographic gratings with certain additives at certain compositions of a thin film (10 μm). Efficient and stable holographic gratings recorded in photopolymers have unique properties, making them attractive for a wide variety of applications, such as 3D displays [34], photonics [35], solar concentrators [36], holographic autocorrelators, laser ultrasonic receivers, ultrasound-modulated optical tomography, holographic optical coherence imaging and surface waveguides [2]. Another potential application of holographic materials is holographic data storage, a technique that might compete in certain niches with other storage technologies [37]. However, the main challenge is to approach an ideal medium that has a high performance at reduced economical costs. It is expected that a proper combination of ILs with an efficient photopolymer would converge to that goal.

While Lin et al. focused their detailed investigation on the chemistry, e.g., employing different compositions based on ILs and PEGDMA as the recording material for thin gratings, we present a comprehensive study of the physical properties of gratings recorded in one of the most efficient composites. Such gratings could originate from a modulation of the refractive-index (phase gratings), of the absorption (amplitude gratings) or even of both of them. In the latter case, we call them mixed gratings. The study includes different recording conditions for a series of thicknesses, a detailed characterization of their type, their angular sensitivity and the corresponding diffraction efficiency, as well as detrimental features, such as the strength of light-induced scattering or a possible dark polymerization.

## 2. Materials and Methods

### 2.1. Sample Preparation

As a starting point for the materials’ preparation, we have chosen one of the compositions described in [24,32,33] for which the highest diffraction efficiency was obtained.

We used the poly-(ethylene glycol)-dimethacrylate (average M${}_{n}$ 550) as a monomer, 1-hydroxylchohexyl phenyl ketone 98% as the UV photoinitiator, polyvinyl acetate (PVAC) as a polymer binder and the ionic liquid 1-butyl-3methylimidazolium tetrafluoroborate (BMIMBF${}_{4}$). All materials were used as received. The samples were prepared from the UV curable emulsion containing 72.5 wt % of the PEGDMA (Sigma Aldrich), 18.0 wt % of the IL (Sigma Aldrich), 8.1 wt % of the PVAC (Alfa Aesar) and 1.4 wt % of the photoinitiator (Alfa Aesar). The mixture with a refractive index of $n_{0}=1.46$ was inserted into glass cells consisting of two glass plates separated by Mylar spacers of various thicknesses ($d_{0}=10,20,50,85,100,125,150\;\mu m$).

### 2.2. Optical Properties

To characterize the optical properties of the samples, absorbance spectra in the UV and visible range were recorded using a CARY5G (Varian) in double-beam configuration at perpendicular incidence. The measured absorbance includes reflection (from the cover plates of glass), absorption and scattering losses.

### 2.3. Holographic Recording (Grating Preparation)

**Figure 1:**
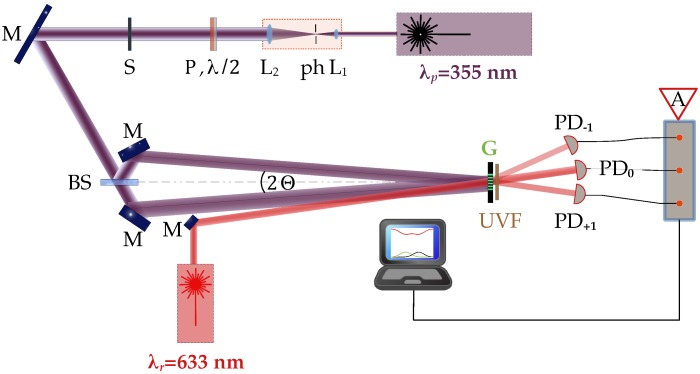
Schematic of the experimental setup. Continuous-wave solid-state laser ($\lambda_{p}=355\;\mathrm{nm}$); L${}_{i}$: lenses; ph: pinhole; P: polarizer; λ/2-plate; S: shutter; M: mirror; BS: beam splitter; G: grating (sample); UVF: UV-filter; PD${}_{s}$: Si-photodiodes for the *s*-th diffraction order; He-Ne laser ($\lambda_{r}=633\;\mathrm{nm}$); A: photoamplifier.

Holographic gratings were recorded by using a standard two-wave mixing setup (Figure 1). Two coherent *s*-polarized expanded beams from a continuous-wave solid-state laser at a wavelength of $\lambda_{p}=355\;\mathrm{nm}$ are superimposed at an intersection angle of $2\Theta \approx 3.5^{\circ }$, resulting in an interference pattern with a grating period of about Λ≈5.8μm. The sample is then placed in the plane of interference so that the two recording beams impinge symmetrically on the sample and generate a transmission grating due to photopolymerization. The recording beams have equal intensities of $I=3.4\;\mathrm{mW}/{\mathrm{cm}}^{2}$ each, the recording times are either $t_{p}=12\;s$ to have comparable conditions to [33] or $t_{p}=90$ s to realize the polymerization rate controlled process. The sample cell is fixed on a computer-controlled piezo-translation stage mounted on a rotation stage.

### 2.4. Readout Techniques

During the recording process, an *s*-polarized low intensity readout beam at $\lambda_{r}=633\;\mathrm{nm}$, a wavelength at which the holographic material is insensitive, diffracts from the developing grating at the first order Bragg angle. The diffracted intensities of the zeroth and first orders are monitored as a function of exposure time.

**Figure 2:**
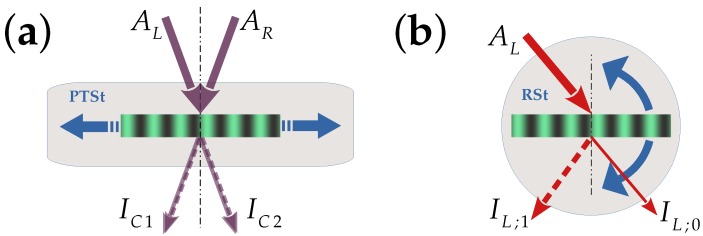
Schematic of the experimental setup for (**a**) a two-beam coupling (2BC) experiment and (**b**) an angular selectivity experiment. PTSt: piezo-translation stage; RSt: rotation stage. $A_{L,R}$ are the amplitudes of the incident beams; $I_{C1,C2}$ the coupled intensities. $I_{L;s}$ are the intensities of the *s*-th-order diffracted beams.

After recording of the grating is finished, two-beam coupling (2BC) experiments are conducted (see Figure 2). The intensity of the beams is reduced by a factor of 100 as compared to recording, i.e., the 2BC measurements are made at very low exposure. The grating is translated parallel to the grating vector $\vec{G}$ for a distance of a few grating spacings, say 25 μm, with the help of the piezo-translation stage. The intensities $I_{C1,C2}$ of the coupled beams as a function of the translation distance are measured. Due to the interference effects of the beams in the grating region, i.e., $I_{C1}=|A_{R;0}+A_{L;1}{|}^{2},I_{C2}={\left|A_{L;0}+A_{R;1}\right|}^{2}$, it is possible to retrieve the following information: (1) the type of grating ((phase grating, amplitude grating or mixed grating)); (2) the magnitude of each of the grating amplitudes; (3) a tentative relative phase between the two grating types; and (4) the relative phase between the recording interference pattern and the grating. Here, $A_{u;s},u=L,R;s=0,1$ are the amplitudes of the left/right (L,R) beams diffracted into the zero/first (0,1) orders. A schematic of the experimental setup is shown in Figure 2a.

An extremely useful quantity relating theories and experimental results is the diffraction efficiency $E_{s}$. It is defined as the diffracted power $P_{s}$ into a certain diffraction order *s* over the incident power *P*:

(1) $$E_{s}=P_{s}/P$$

More often, when losses can be neglected, the more convenient relative diffraction efficiency $\eta_{s}$ is used instead, where the incident power is replaced by the sum of the powers diffracted into each order, $\eta_{s}=P_{s}/\sum_{j}P_{j}$. We will use the latter whenever it is applicable.

The angular dependence of the diffraction efficiency, also called “rocking curves”, of the gratings is obtained using a low intensity He-Ne probe beam. This experiment is important in order to reveal the effective grating thickness *d*, which might differ from the geometrical thickness $d_{0}$, e.g., due to polymer shrinkage. For a final evaluation of the 2BC parameters, the effective thickness is decisive. Furthermore, while the 2BC experiment yields information only at the Bragg angle, i.e., a single point, the information obtained via the angular dependence is complementary and more comprehensive in the sense that it allows one to distinguish different regimes of overcoupling. Figure 2b presents a sketch of the setup for measuring the angular dependence of the diffraction efficiency.

## 3. Experimental Results

### 3.1. Optical Quality and Morphology of the Samples

**Figure 3:**
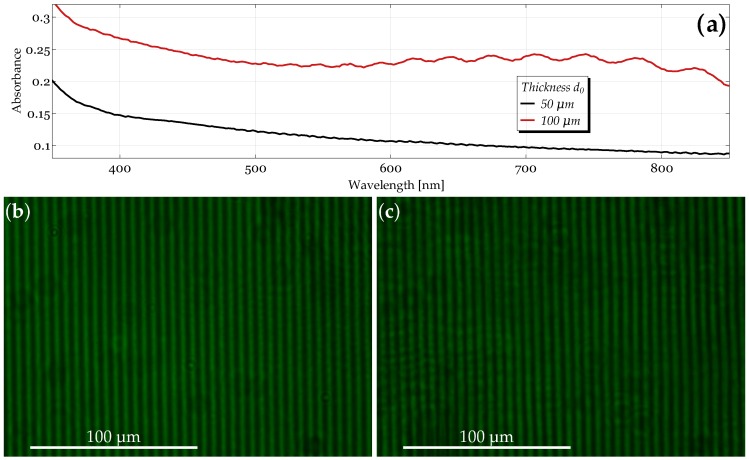
Characterization of the samples’ optical quality for $d_{0}=50$ and 100μm. (**a**) Absorbance as a function of wavelength in the UV-Vis range. The data include contributions from reflection (independent of $d_{0}$), absorption and scattering losses. The morphology of the gratings visualized by polarization optical microscope (POM) (λ=546 nm) for (**b**) $d_{0}=50$ and (**c**) 100μm, respectively.

To characterize the overall optical quality of the samples, absorbance spectra were measured in the UV-Vis range. The absorbance, i.e., the logarithmic ratio of the transmitted over the incoming power, is shown in Figure 3a for sample thicknesses of $d_{0}=50\;\mathrm{and}\;100\;\mu$m in the wavelength range from 350–850 nm. In addition, the morphology of the gratings with a spacing around six microns is easily visualized by using a polarization optical microscope (POM) with a resolution of about half a micrometer. In Figure 3b,c, POM images of ionic liquid composite gratings are shown. POM of the gratings for other thicknesses can be found in the Supplementary Materials.

While reflection losses are independent of the sample thickness and are in the range of a few percent for our samples, absorption, as well as scattering are expected to increase for thicker samples. However, it can be clearly seen that the sample with $d_{0}=100\;\mu$m shows enhanced absorbance with an unusual structure in the red wavelength range. We attribute this to the strong wide angular holographic scattering, i.e., the light does not reach the detector. Absorption is responsible for losses only in the UV, whereas over the whole visible range, light-induced (holographic) scattering is the major reason for losses up to 40% in total. The POM images confirm the recording of homogeneous periodic structures with a spacing of about Λ≈5.9μm.

### 3.2. Holographic Recording

**Figure 4:**
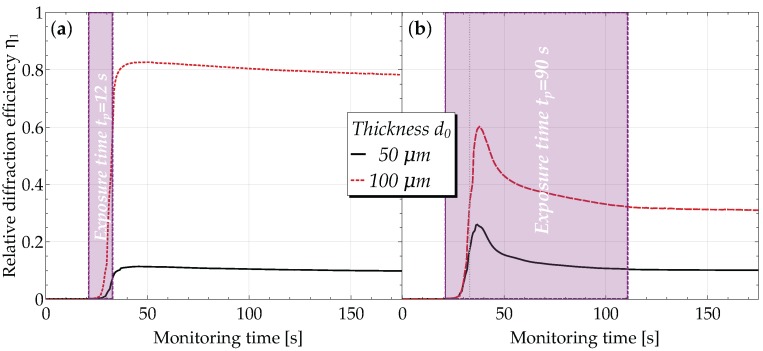
The +1st order relative diffraction efficiencies at the Bragg angle as a function of time for samples with a recording exposure of (**a**) $t_{p}=12$ s and (**b**) $t_{p}=90$ s, respectively. The readout wavelength was $\lambda_{r}=633$ nm.

During the recording process, the kinetics of grating evolution was probed by measuring the diffraction efficiency $\eta_{1}$ as function of time at $\lambda_{r}=633\;\mathrm{nm}$ (Figure 4). The probe beam was adjusted to enter the grating at the corresponding Bragg angle ($\Theta_{r}\approx 3.18^{\circ }$).

For gratings with a recording time $t_{p}=12$ s, $\eta_{1}$ steeply increases even after the UV exposure stopped, reaches a maximum within a few seconds and slowly approaches saturation at slightly decreased values.

Similarly, the gratings recorded for $t_{p}=90$ s first show an increase of $\eta_{1}$ as a function of time, then pass a maximum followed by a rapid decrease for some ten seconds. Finally, they also approach their saturation value.

### 3.3. Two-Beam Coupling

**Figure 5:**
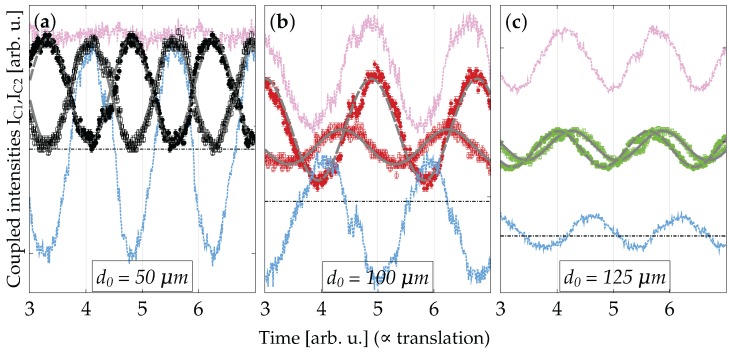
Experimental data for the coupled intensities as a function of time during ramping up the voltage to the piezo-translation stage. Time is directly proportional to the traveled distance along the grating vector. $t_{p}=12$ s, $\lambda_{r}=355$ nm and: (**a**) $d_{0}=50\;\mu m$ (black symbols); (**b**) $d_{0}=100\;\mu m$ (red symbols); (**c**) 125μm (green symbols). Grey dashed lines are fits according to the theory (cf. Section 4.3). The pink and blue lines are the sum (Σ) and the difference (Δ) of the coupled intensities, respectively (see the text); dash-dotted horizontal lines denote zero intensity.

Tow-beam coupling experiments were performed immediately after recording using UV beams at $\lambda_{r}=355$ nm. As an example, the coupled intensities $I_{C1},I_{C2}$ as a function of the grating translation for three nominal thicknesses ($d_{0}=50,100,125\;\mu m$) and a recording time of $t_{p}=12$ s are shown in Figure 5. Similar experiments were conducted for $t_{p}=90\;s$ (not shown here).

To get a first qualitative insight into the grating type behavior, we employ the small signal approximation used in [38]. Assuming equal incoming intensities, as is the case for our experiments, we determine the dominant grating type by computing the sum $\Sigma =I_{C1}+I_{C2}$ and the difference $\Delta =I_{C1}-I_{C2}$ of the coupled intensities. For Σ≈const., i.e., $I_{C1}$ and $I_{C2}$ are shifted to each other by *π*, a dominant refractive-index grating (phase grating) is identified. For Δ≈const., i.e., $I_{C1}$ and $I_{C2}$ in phase, the absorption grating prevails. When inspecting the graphs of Figure 5a–c, we can see a clear change of the behavior: for a thin grating (Figure 5a), the refractive-index grating is dominant, whereas for, e.g., $d_{0}=100\;\mu m$, absorptive, as well as refractive-index modulation occurs (mixed grating). Different amplitudes $I_{max}-I_{min}$ for $I_{C1}$ and $I_{C2}$, respectively, point towards a relative phase shift φ≠sπ between the components (Figure 5b), provided similar input amplitudes, which is the case here. Finally, for an even thicker grating, the coupled intensities are close to in-phase (Figure 5c), indicating dominant extinctive behavior. A careful quantitative evaluation will be performed in Section 4.3.

### 3.4. Angular Dependence of the Diffraction Efficiency

**Figure 6:**
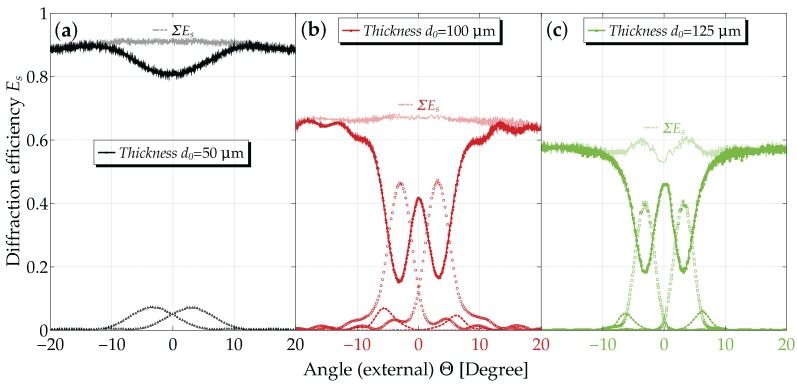
Diffraction efficiency after saturation for a recording time of $t_{p}=12$. $E_{0,\pm 1,\pm 2}$ as a function of the angle of incidence for: (**a**) $d_{0}=50\;\mu m$; (**b**) $d_{0}=100\;\mu m$; and (**c**) $d_{0}=125\;\mu m$. Zero diffraction orders are indicated by filled symbols, first diffraction orders by open symbols and second orders by dotted lines. The latter are invisible for $d_{0}=50\;\mu$m because of their low values. In addition, for each thickness value, the sum of all diffraction orders $\sum_{s=-2,-1\ldots +2}E_{s}$ is shown as a faint line. The rapid oscillations are interference fringes due to the plane-parallel glass covers.

The angular dependencies of the diffraction efficiency $E_{s}$ for a series of thicknesses and for $t_{p}=12\;s$ are presented in Figure 6. Measurements are available also for other thicknesses (as well as for $t_{p}=90\;s$) and are used for data evaluation; see also the Supplementary Materials.

The reduction of $E_{s}$ to values <1 relates to the mean absorption, light-induced scattering, as well as reflection losses. Furthermore, we point out five observations here that will be important for proper modeling in the next section:

1. in the vicinity of Θ=0 (normal incidence), three diffraction orders (0,±1) show considerable intensities, in particular for the thinner samples; for larger grating thicknesses ($d_{0}≳100\;\mu$m), in addition, second order diffraction peaks are increasingly important.
2. the usual oscillatory structure of $E_{\pm 1}$ near the Bragg peak is less pronounced, i.e., minima are observable, but are not zero (cf. $d_{0}=100\;\mu$m).
3. the sum of all diffracted orders, shown as a faint line, has a distinct structure (“double-well”) in its angular dependence for $d_{0}=125\;\mu$m rather than the usually expected smooth behavior (e.g., $d_{0}=100\;\mu$m); we emphasize that in these former cases, it is mandatory to disregard the use of the relative diffraction efficiency $\eta_{s}$!
4. the dependence of $E_{1}$ on the nominal grating thickness $d_{0}$ at the Bragg angle is non-monotonous. This is consistent with the observations made for its time dependence of Section 3.2 (not shown; cf. Supplementary Materials).
5. the maximum diffraction efficiency $E_{\pm 1}$ is about 45% ($\eta_{\pm 1}\approx 70\%$) for $d_{0}=100\;\mu m$, which is a much higher value than reported so far [33] in polymer-ionic liquid composites.

Another important technological issue is the long-term stability of recorded gratings. We measured the angular dependence of *η* from time to time over about two months, and the rocking curves remained the same. This indicates that the gratings are stable for all samples, independent of thickness and exposure time, after saturation was reached. Similar results were obtained for a recording time of $t_{p}=90$ s.

### 3.5. Light-Induced Scattering

Finally, we monitored the scattering patterns visualized on a screen behind the sample using a digital camera.

**Figure 7:**
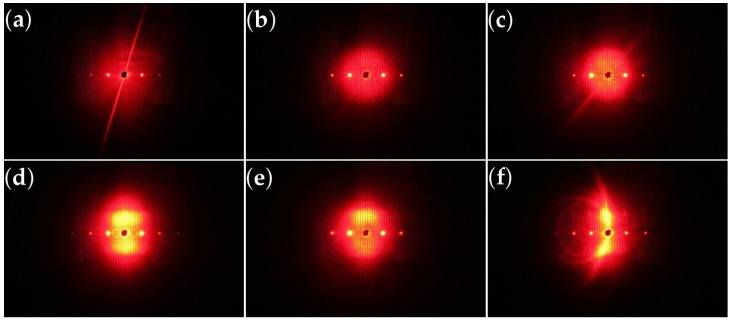
HS: Far-field light-induced scattering patterns on a screen behind the sample. Photos were taken after recording when the sample is illuminated with the probe He-Ne laser beam at the Bragg angle for $\lambda_{r}$. The central black spot is to mask the transmitted beam on the screen to avoid overexposure of the photo taken. $t_{p}=12\;s$ and: (**a**) $d_{0}=20\;\mu m$; (**b**) $d_{0}=50\;\mu m$; (**c**) $d_{0}=85\;\mu m$; (**d**) $d_{0}=125\;\mu m$ or $t_{p}=90\;s$; and (**e**) $d_{0}=125\;\mu m$, respectively. In (**f**), we show diffraction cones (closed rings) for $d_{0}=125\;\mu m$, $t_{p}=12\;s$ due to the presence of parasitic gratings (PGs) (see the text) for an off-Bragg angle of $\theta_{r}=16^{\circ }$ [39].

Figure 7 shows the diffraction patterns of samples with different thicknesses (a–d), or exposure times (d,e) at the first order Bragg angle for the readout wavelength $\lambda_{r}$. The patterns include different diffraction orders from the elementary grating in addition to a wide range of diffuse scattered light centered around the transmitted, i.e., zero-order beam (masked black spot). It is obvious and expected that thicker samples exhibit stronger holographic scattering. Comparing samples of the same thickness (cf. Figure 7d,e) with different exposure times, we find that longer exposure results in less pronounced scattering. Moreover, Figure 7f additionally shows conical scattering of the input beam for an off-Bragg angle, which confirms the presence of parasitic gratings (PGs) in the samples.

Usually, holographic recording is accompanied with a gradual increase of light-induced scattering. The latter is due to the formation of PGs, which are generated simultaneously with the desired elementary grating. These PGs appear as long as there is any source of seed scatterers present. In addition, a sufficiently effective amplification mechanism for that scattered radiation, e.g., the photorefractive effect in our case, is required. The seed scatterers might be defects, imperfections and/or optical inhomogeneities within the medium. Therefore, light-induced scattering is a general phenomenon that occurs in virtually all media for which refractive-index changes occur when irradiated with a coherent beam [39,40,41,42,43,44,45]. The presence of these PGs hinders the applicability of elementary gratings by corrupting the optical quality of the system, e.g., by reducing the signal to noise ratio. However, light-induced scattering can also be exploited in a wide variety of applications, such as optical limiting, motion detection and phase conjugation mirrors; see, e.g., [46].

## 4. Modeling and Data Evaluation

### 4.1. Recording a Holographic Grating

Recording a holographic grating in a photosensitive material is essentially connected to an absorptive process: in the photopolymer composites under investigation here, absorbed photons trigger polymerization in illuminated regions. The polymerization causes a change and/or modulation of the density, which results in a refractive-index modulation proportional to the exposure Q=It, i.e., the product of intensity and time. There are sophisticated theories to predict the temporal evolution of the diffraction efficiency, e.g., [47]. For our system, the nonlocal-response diffusion model of holographic recording might be suitable, a detailed theory developed in a series of papers by Sheridan and coworkers [48,49,50,51,52]. This includes also the implications and the role of absorption during the recording process, among them a change of the spatial profile of the recorded grating during exposure, a feature already discovered in the 1970s [53] in photochromic materials. We focus on another peculiarity: as light is absorbed, the intensity of the interference pattern decreases through the thickness of the holographic medium, and this inevitably leads to a depth-dependent refractive-index and absorption modulation called tapering [54,55,56,57]. Important consequences are reduced (averaged) diffraction efficiency and altered angular response.

### 4.2. Transmission Grating Geometry

**Figure 8:**
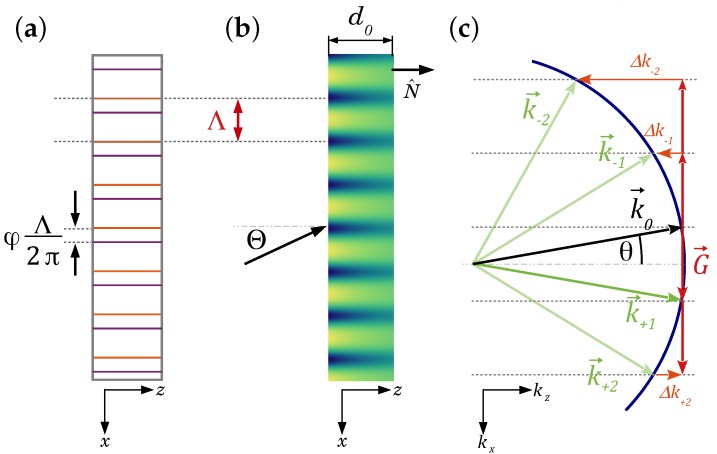
(**a**) Schematic for 2BC from mixed gratings with a phase shift *φ*. Orange and purple lines denote the maxima of the refractive index and the absorption grating, respectively (cf. Equations (3) and (4)). (**b**) Diffraction geometry of a one-dimensional in-plane ($\hat{N}\in$ plane of incidence) grating with attenuation along the depth *z*. Λ=2π/G is the grating spacing; the incident wavevector reads $\vec{q}=2\pi /\lambda \left(\sin\Theta ,0,\cos\Theta \right)$, where *λ* is the wavelength of the light in free space and Θ the angle of incidence outside the medium. $\hat{N}$ is the sample surface normal. (**c**) Diffraction geometry in reciprocal space. The grating vector is $\vec{G}=\left(G,0,0\right)$; ${\vec{k}}_{s}$ is the real part of the wavevector of the *s*-th order diffracted beam in the medium, and thus, $|{\vec{k}}_{s}|=2\pi n_{0}^{\prime }/\lambda$, as well as $\sin\Theta =n_{0}^{\prime }\sin\theta$. $\Delta k_{s}$ is the corresponding momentum mismatch when the Bragg condition is not fulfilled. Here, five-wave coupling is shown in the case that the Bragg condition is obeyed for the +1st order; the blue circle indicates the Ewald sphere.

We start the discussion with defining the geometry and the profile of the holographic grating, i.e., the refractive-index profile under investigation (see Figure 8).

The complex refractive index shall be periodic along the *x*-direction and might have a smooth dependence along the *z*-direction (see Figure 8). Furthermore, we assume unslanted transmission geometry, i.e., the sample surface normal is perpendicular to $\vec{G}$. Finally, the wave vector $\vec{q}$ of the incident wave is in the plane x−z (in-plane diffraction). Thus, in the most general form, we expand the complex refractive index $\tilde{n}$ in a one-dimensional Fourier series [57]:

(2) $$\tilde{n}\left(x,z\right)=n_{0}^{\prime }-ın_{0}^{\prime \prime }+\sum_{s=1}^{\infty }\left[n_{s}^{\prime }\left(z\right)\cos(sGx)-ın_{s}^{\prime \prime }\left(z\right)\cos\left(sGx+\varphi_{s}\right)\right].$$

Here, $n_{0}^{\prime }$ is the average refractive index; $n_{0}^{\prime \prime }$ relates to the mean absorption constant by $\alpha_{0}=2\left|\vec{k}\right|n_{0}^{\prime \prime }$; $n_{s}^{\prime }\left(z\right)$ and $ın_{s}^{\prime \prime }\left(z\right)$ are the depth-dependent real and imaginary parts of the Fourier components, respectively; and $\varphi_{s}$ accounts for a tentative relative phase between the components of the refractive index and the absorption. These parameters can be determined experimentally by 2BC experiments in principle (see Section 4.3). Of course, Equation (2) is much too general for our considerations, and we use dedicated simplified versions.

### 4.3. Two-Beam Coupling

Two-beam coupling experiments are basically interferometric measurements, which allow one to determine the amplitudes and phases of waves that are Bragg diffracted from a (complex) grating. By measuring the intensities of the interfering beams (see Figure 2a), the decisive parameters of the corresponding grating can be determined. To evaluate our experimental data shown in Figure 5, we simplify Equation (2) as (with $n_{0}^{\prime }\to n_{0}$):

(3) $$\begin{array}{ccc} n(x) & = & n_{0}+{\bar{n}}_{1}\cos\left(Gx+\phi_{n}\right)\;\mathrm{and}/\mathrm{or} \end{array}$$

(4) $$\begin{array}{ccc} \alpha (x) & = & \alpha_{0}+{\bar{\alpha }}_{1}\cos\left(Gx+\phi_{\alpha }\right). \end{array}$$

Higher Fourier components are simply disregarded; the imaginary part of the refractive index (absorption) results in loss of diffracted intensity (Lambert–Beer’s law) and its modulation $n_{1}^{\prime \prime }\propto \alpha_{1}$ to an amplitude grating. A relative phase shift $\varphi =\phi_{\alpha }-\phi_{n}$ between the two types of gratings is considered, as well. As already mentioned above, we call such gratings mixed gratings. As the interfering beams are assumed to fulfill the Bragg condition, a depth dependence of $n_{1}\left(z\right)$ (and $\alpha_{1}\left(z\right)$) leads to an averaged valued ${\bar{n}}_{1}=1/d\int_{0}^{d}n_{1}\left(z\right)dz$. In common interferometric experiments, it is assumed that by translating the analyzer grating, intensity oscillations that are out-of-phase occur [58]. This is a consequence of energy conservation if the beam splitter is lossless. Contrary to this result, pure absorption gratings used as beam splitters would result in intensity oscillations that are in phase.

To evaluate $n_{1},\alpha_{1},\varphi$ for the mixed gratings, we employ the procedure as described in [59,60]: the oscillatory functions $I_{C1,C2}=a_{1,2}+b_{1,2}\cos\left(Gx+c_{1,2}\right)$ are fitted to the experimentally-measured coupled intensities and then related to the physical quantities.

The beam coupling data were evaluated for a series of thicknesses for both recording times (see Figure 5 for $t_{p}=12\;s$). For a sound evaluation, it is necessary to use further information, such as the incoming intensities and the thickness, the latter of which we could take from the rocking-curve measurements in Section 3.4 and Section 4.5. However, ambiguities in the evaluation due to overcoupling, i.e., for ${\bar{n}}_{1}d/\lambda \cos\theta_{B}>1/2$, still remain [59]. Different sets of parameters fit the data equally well. We therefore can only determine them reliably for the thinner samples, say $d_{0}\leq 50\;\mu m$ (see Table 1).

### 4.4. Diffraction from Holographic Gratings: The Readout Process

When inspecting the measured angular dependence of the diffraction efficiency for various thicknesses (Figure 6), we find two remarkable properties already addressed above: (1) near normal incidence, at least three waves couple for the thinner samples, while this is not the case for the thicker ones; the latter in turn even show higher harmonics, i.e., a non-sinusoidal refractive index; (2) the oscillatory side peak structure clearly shows nonzero minima, in particular for the thicker samples. This indicates a depth-attenuated refractive-index profile [54,61]. Both of these features have to be accounted for in a theory to properly describe our experimental results. This issue will be discussed in what follows.

### 4.5. Multi-Wave Coupling of Mixed, Shifted Refractive-Index and Absorption Gratings, Including an Attenuation along the Sample Depth

In their seminal paper in 1980 [62], Moharam and Gaylord presented the rigorous-coupled wave analysis (RCWA), which allowed calculating the diffraction properties of a sinusoidal phase grating in a rigorous way, i.e., considering an arbitrary number of coupled waves, covering all types of diffraction regimes. Thus, the RCWA would be the proper theory to account for (1) and to extract $n_{1}^{\prime },n_{1}^{\prime \prime }$ from our data provided that attenuation of the recorded pattern along the depth is negligible. This would be a good approximation for the thinner samples only. For the thicker samples, we definitely must account for such an attenuation. Experiments show that an exponential decay is a reasonable assumption, $n_{1}\left(z\right)=n_{1}^{0}\exp\left(-z/L\right)$, with *L* being the characteristic decay length. A similar dependence for the absorption modulation holds. $n_{1}^{0}$ and the average refractive-index modulation ${\bar{n}}_{1}$ of Equation (3) are related by ${\bar{n}}_{1}=n_{1}^{0}L/d\left[1-\exp(-d/L\right)]$.

There are plenty of theories to describe diffraction under two-wave coupling conditions without attenuation along the depth (e.g., [63,64,65]), maybe the most famous and extensively quoted one by Kogelnik [66]. For in-phase mixed gratings, the contributions of both components for the diffraction efficiency $\eta_{1}$ simply add up [66], i.e.,

(5) $$\eta_{1}=\left[\sin^{2}\left(\frac{n_{1}\pi d}{\lambda \cos\theta_{B}}\right)+\sinh^{2}\left(\frac{\alpha_{1}d}{2\cos\theta_{B}}\right)\right]e^{-\frac{2\alpha_{0}d}{\cos\theta_{B}}}$$

when the Bragg condition is fulfilled.

In a more advanced analysis a relative phase shift *φ* between the refractive-index and the amplitude grating is retained and might, e.g., lead to different values for $\eta_{+1}$ and $\eta_{-1}$. Unraveling those contributions can be done by performing 2BC experiments [38,59,60] (see Section 4.3), although diffraction experiments reveal certain signatures of such gratings, as well [67,68,69]. One disadvantage of 2BC experiments is that information is taken only at the Bragg angle. Information about the thickness or even a decay of the grating along the thickness cannot be obtained without additional measurements. To obtain the characteristic parameters $n_{1},\alpha_{1},\varphi ,d$ from the measured angular dependence data (Figure 6), we set up a multi-wave coupling theory for shifted mixed gratings with an attenuation along the grating depth. The tedious task of including such a behavior into the RCWA (for pure phase gratings) was performed in [61] by dividing the hologram into a large number of layers along the sample depth, each of them treated by the RCWA and considering the boundary conditions between subsequent layers. We adopt a simpler approach: a coupled-wave ansatz is inserted into the wave equation, which leads to a system of differential equations to be solved (numerically) using appropriate boundary conditions. We start with the wave equation:

(6) $$\left(\nabla^{2}+q^{2}\right)E\left(x,z\right)=0,$$

where [67]:

(7) $$q^{2}=\beta \left(\beta -2ı\alpha_{0}+2\kappa_{1}^{+}e^{ıGx}e^{ı\phi_{n}}+2\kappa_{1}^{-}e^{-ıGx}e^{-ı\phi_{n}}+\kappa_{2}e^{ı2Gx}e^{ı\phi_{n}}\right).$$

Here, $\beta =n_{0}2\pi /\lambda$ is the mean propagation constant, and $\kappa_{1}^{\pm }\left(z\right)=\left[n_{1}\left(z\right)\pi /\lambda -ı\alpha_{1}\left(z\right)e^{\pm ı\varphi }/2\right]$, $\kappa_{2}=n_{2}\left(z\right)\pi /\lambda$ are the (generally complex) depth-dependent coupling constants. We consider only the first and second grating harmonics. We assume that for the latter, only the refractive-index modulation is of relevance. E(x,z) is the (s-polarized) electric field amplitude, which can be represented in the grating region by a coupled-wave ansatz:

(8) $$E\left(x,z\right)=\sum_{j}S_{j}\left(z\right)e^{-ı{\vec{k}}_{j}\cdot \vec{x}},$$

and $S_{j}\left(z\right)$ are the diffraction amplitudes to be determined. The Floquet condition requires:

(9) $${\vec{k}}_{j}={\vec{k}}_{0}+j\vec{G}-\Delta k_{j}\hat{N},$$

with the (scalar) mismatch parameter:

(10) $$\Delta k_{j}=\beta \left[\cos\theta -\sqrt{1-{\left(\sin\theta +jG/\beta \right)}^{2}}\right].$$

*θ* denotes the angle (measured in the medium) between the sample surface normal $\hat{N}$ and the internal wavevector ${\vec{k}}_{0}$ of the incident beam. By inserting Equations (7)–(10) into the wave Equation (6), neglecting second order derivatives and sorting the terms according to their propagation direction, the coupled differential equations to be solved for $S_{j}\left(z\right)$ with the boundary conditions $S_{0}\left(z=0\right)=1$ and $S_{j\neq 0}\left(z=0\right)=0$ read:

(11) $$\begin{array}{ccc} ık_{j,z}\frac{\partial S_{j}\left(z\right)}{\partial z} & = & \beta \left[\kappa^{+}\left(z\right)S_{j+1}\left(z\right)e^{ı(\Delta k_{j+1}-\Delta k_{j})z}+\kappa^{-}\left(z\right)S_{j-1}\left(z\right)e^{ı(\Delta k_{j-1}-\Delta k_{j})z}+\right.\\ & + & \left.\kappa_{2}\left(z\right)\left(S_{j+2}\left(z\right)e^{ı(\Delta k_{j+2}-\Delta k_{j})z}+S_{j-2}\left(z\right)e^{ı(\Delta k_{j-2}-\Delta k_{j})z}\right)-ı\alpha_{0}S_{j}\left(z\right)\right]. \end{array}$$

The *j*-th order diffraction efficiency $\eta_{j}$ is then obtained by $\eta_{j}={\left|S_{j}\left(z=d\right)\right|}^{2}k_{j,z}/k_{0,z}$.

**Figure 9:**
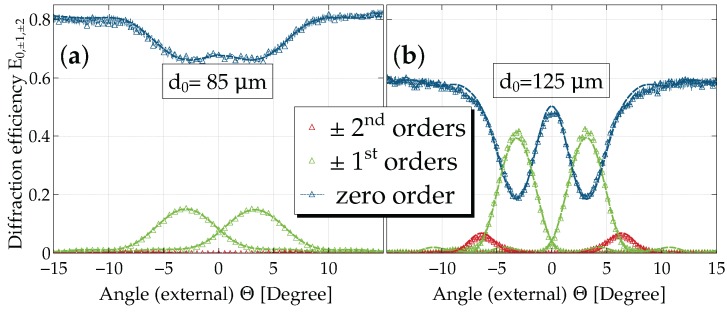
Angular dependencies of $E_{0,\pm 1,\pm 2}$ for a grating with (**a**) $d_{0}=85\;\mu m$ and (**b**) $d_{0}=125\;\mu m$, $t_{p}=12\;s$. Dashed lines are fits to the data based on numerically solving Equation (11).

For an evaluation of the grating parameters from the angular dependence of the diffraction efficiencies, we solved the coupled-wave Equation (11) numerically for j=−2,…,+2, i.e., five-wave coupling. As discussed above, we assumed an exponential decay of the grating along the depth and further kept $n_{1},\alpha_{1},\varphi ,n_{2},d,L$ as free fitting parameters. Reflection losses of both glass covers were accounted for by using Fresnel’s equations. While the data up to about $d_{0}=85\;\mu m$ can be fitted very nicely by the model, the thickness region where substantial holographic scattering occurs ($d_{0}=100,125\;\mu m$) is tedious to fit and much less satisfactory (see the $\chi^{2}$-value in Table 2). Figure 9 shows the measured data and the fitted curves for $d_{0}=85\;\mu m$ and $d_{0}=125\;\mu m$, respectively.

In Table 2, an overview of the physical parameters determined by fitting the angular dependence to the diffraction efficiency for various thicknesses is given together with the dimensionless Klein–Cooke parameter $Q=2\pi \lambda d/(n_{0}\Lambda^{2})$. A more elaborate analysis of the diffraction regimes depending not only on the thickness, but also on the refractive-index modulation is provided in the Supplementary Materials [70,71,72] .

## 5. Discussion

**Figure 10:**
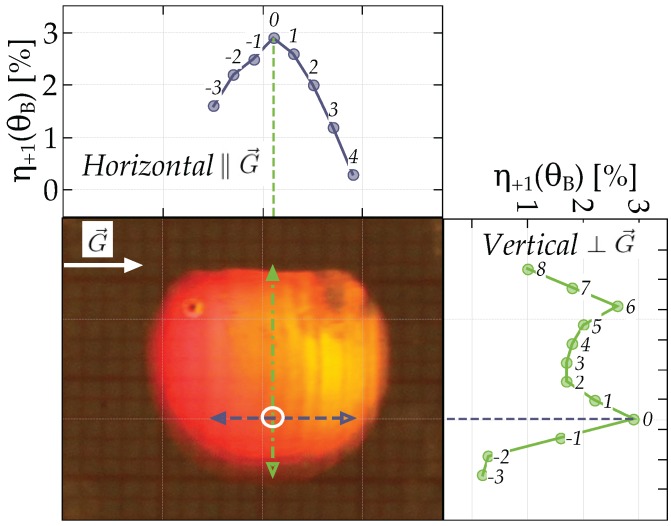
Diffraction efficiency $\eta_{+1}\left(\theta_{B}\right)$ at the Bragg angle across the grating area, i.e., $∥\vec{G}$ and $⊥\vec{G}$, for $d_{0}=20\;\mu m$ and $t_{p}=12$ s. The lower left is a photo of the hologram (in reflection). Labels give the displacement from the zero position (maximum diffraction efficiency) of the beam in millimeters.

In general, the gratings are quite inhomogeneous across the sample area. As an example, we show the diffraction efficiency $\eta_{+1}\left(\theta_{B}\right)$ for a cross-section along the grating vector and perpendicular to it, respectively (Figure 10). The hologram, i.e., the recording area, is of a circular shape (segment of a circle) with about 10 mm in diameter.

In all of our experiments, the region with the highest diffraction efficiencies, i.e., best quality, for each of the investigated samples was determined as above.

### 5.1. Recording

The temporal evolution of the diffraction efficiencies during recording, and for a time span of at least one minute after having switched off the interference pattern, is remarkable when comparing different exposure times.

For gratings recorded for $t_{p}=12\;s$, the value of the diffraction efficiency continues to increase for a few seconds after the termination of the holographic exposure (Figure 4a). The increase of the diffraction efficiency for the different sample thicknesses is non-monotonous and in the range from 36% (125μm) to 65% (150μm). This increase occurs within a short time of about 4 s. However, it is reported in the literature that polymerization processes continue for days in the dark after the termination of the irradiation [73]. This fast dark polymerization may be useful for applications with polymeric biomaterials where the ‘shadow’ and depth of cure are critical [74,75]. When recording for $t_{p}=90\;s$ (Figure 4b), the diffraction efficiency increases rapidly, as well followed by a slow decrease until it stabilizes just before switching off the recording beams.

As discussed in [24], this characteristic time dependence can be explained as follows: having photoinitiators in our composite, it goes without saying that the polymerization process occurs more rapidly in the brighter regions of the intensity distribution. This in turn gives rise to monomer diffusion from dark regions (high monomer concentration) into bright regions, and a density modulation, viz. a grating, builds up. Now, due to the presence of ILs, two driving forces for the ongoing polymerization are competing: (1) for short recording times, a diffusion rate controlled polymerization takes place; this even leads to an enhancement of the refractive-index modulation [33]; (2) for longer recording times, we end up with a polymerization rate controlled process [33], which causes a decrease of the diffraction efficiency after some time.

Finally, focusing on the time dependence of $\eta_{1}$ after having switched off the recording beams, we notice an increase. Therefore, dark diffusion of monomers occurs (in the case of short recording times), which may be attributed to the diffusion-controlled polymerization [24].

### 5.2. Two-Beam Coupling

Due to the discussed ambiguity for the evaluation of the parameters for larger thicknesses discussed above, we remain limited to qualitative reasoning (see Section 3.3). A comparison of the 2BC parameters for the thinner samples and the results obtained by the rocking-curve analysis yields that they are well within the same magnitude for the refractive-index modulation (${\bar{n}}_{1}\approx 2\times 10^{-3},1\times 10^{-3}$ for $d_{0}=20,50\;\mu m$). The modulation of the losses ${\bar{\alpha }}_{1}$, however, is about an order of magnitude larger for the 2BC evaluation than for the angular dependence. This can naturally be explained by the fact that the 2BC experiments are performed at the UV recording wavelength $\lambda_{p}=355$ nm, whereas the rocking-curves are measured at $\lambda_{r}=633$ nm. Yet, the phase grating part dominates the experimental response. For the intermediate thickness region ($100\;\mu m<d_{0}<125\;\mu m$), an extremely strong component of the extinction grating in addition to the phase grating, shifted by *φ* with respect to each other, contributes to the interferometric signal. We attribute the appearance of a pronounced extinction at these thicknesses to the massive onset of holographic scattering; see Section Holographic Scattering below. A similar observation was made in holographic polymer dispersed liquid crystals [76] and polymer-nanocomposite materials [44].

### 5.3. Angular Dependence of the Diffraction Efficiency

**Figure 11:**
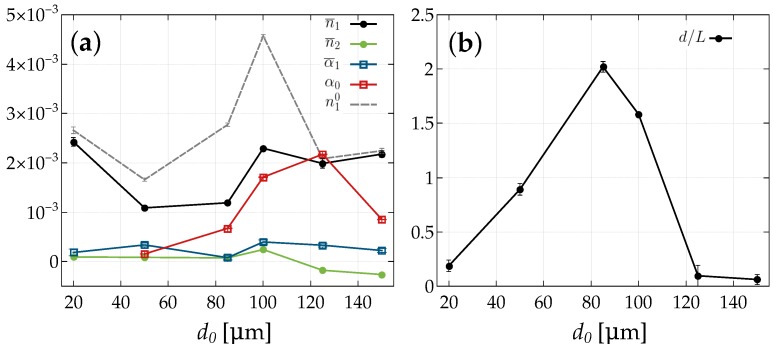
Important parameters as a function of thickness: (**a**) ${\bar{n}}_{1},{\bar{n}}_{2},{\bar{\alpha }}_{1},\alpha_{0},n_{1}^{0}$ and (**b**) the thickness over the decay length.

Inspecting Figure 11 and Table 2, we conclude that gratings with a thickness of up to 100 μm can be regarded as a limit for a simple phase grating. For this thickness regime, we find that:

- the diffraction efficiency, as well as the mean extinction increase with increasing grating thickness
- the sum of the intensities of all diffracted orders adds up to the mean extinction values for all of the angular dependence, and thus
- the grating can be regarded to be dominantly of the phase grating type
- the use of the relative diffraction efficiency $\eta_{s}$ therefore is justified.

On the contrary, we see that for larger thicknesses, in particular for the $d_{0}=125\;\mu$m sample, a behavior, which is characteristic for absorption (here: extinction) or/and mixed gratings, occurs: the sum of intensities of the diffracted orders deviates from the mean extinction value. In these cases, we have to consider the absolute diffraction efficiency $E_{s}$. Moreover, the second order diffracted intensities become prominent. Furthermore, it is interesting to note that the mean extinction is lower for the thickest grating as compared to the one with $d_{0}=125\;\mu$m (see Table 2), a feature that needs to be further investigated. Thus, we learn that this intermediate thickness regime is most unfavorable for improving the diffraction efficiency.

Another interesting observation is that the Fourier coefficient ${\bar{n}}_{2}$ of the second order diffracted beams is negative for the thicker samples, i.e., the (real part of the) refractive-index $n\left(x\right)\propto |{\bar{n}}_{1}\left|\cos\left(Gx\right)-\right|{\bar{n}}_{2}|\cos\left(2Gx\right)+\ldots$. It seems surprising that it is obviously possible to solve the phase problem of scattering in this particular case. However, mostly due to the parameter choice λ/Λ≪1, multi-wave-coupling (this is: multi-wave-interference) occurs for waves of similar amplitude. Thus, employing the five-wave coupling model enables us to extract the (relative) sign of the Fourier coefficient for the first and second orders because reference beams are available. In crystallography, it was shown that by allowing simultaneous multiple diffraction, a solution to the phase problem can be obtained, as well [77,78].

#### Holographic Scattering

**Figure 12:**
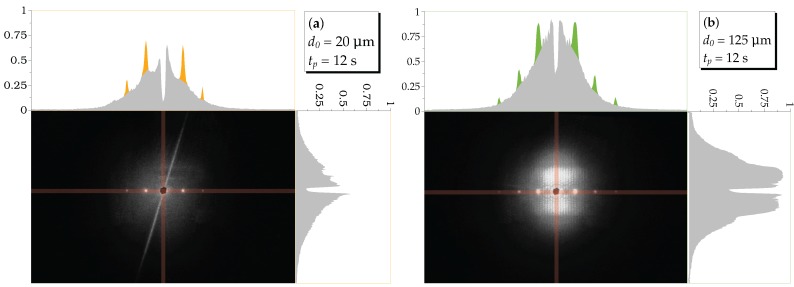
Profiles of the diffraction on top of the holographic scattering patterns for (**a**) $d_{0}=20\;\mu m$ and (**b**) $d_{0}=125\;\mu m$. The scale relates to the intensity averaged over a stripe of 12 pixels (indicated by the faint red color in the photo). Gray shaded areas are due to holographic scattering (PGs), the bright peaks due to diffraction from the elementary gratings. Both photographs were taken with the same exposure time (15 s) and intensity of the readout beam.

The two-dimensional scattering patterns were analyzed along their vertical and horizontal directions through the zeroth diffraction order from where the scattered light originates (see Figure 12). This allows one to get a quantitative measure of the scattering distribution. The images were taken at the Bragg angle for the readout beam, the first diffraction order for $\lambda_{r}$. We do the analysis exemplarily for $d_{0}=20\;\mu m$ and $d_{0}=125\;\mu m$; $t_{p}=12$ s shown in Figure 7a,d; the corresponding results are given in Figure 12a,b, respectively.

The distribution of scattered light from PGs around the zero order diffracted beam has a Gaussian-shaped background (gray filling). The peaks on top of this background (colored filling) are due to the ±1st, ±2nd diffraction orders from the elementary gratings. Note that the sudden drop of the intensity at the position of the transmitted beam is due to the beamstop placed on the screen. The scattered intensity strongly depends on the grating thickness. Light-induced scattering is weak and negligible for thin samples (Figure 12a), whereas for thicker samples, e.g., (Figure 12b) it becomes prominent. Starting from thicknesses of about $d_{0}≳85\;\mu$m, light-induced scattering will be essential, i.e., detrimental for applications, such as data storage. We can therefore consider this thickness as a limit for preparing proper gratings. This is fully consistent with the evaluation of the rocking-curve data when the amplitude of the extinction gratings becomes important and the decay of the grating along the thickness is vital (see Table 2 and Figure 11b).

When reconstructing the elementary grating at the Bragg angle for $\lambda_{p}$, at the same time, all recorded PGs fulfill their corresponding Bragg conditions, as well. In this case, the parasitic scattering centered around the forward beam is maximized. A simple method to reduce this background is to employ a different readout wavelength $\lambda_{r}\neq \lambda_{p}$ (as done in this study) [79]. Then, the elementary grating is still completely reconstructed, but for the PGs, the Bragg condition is fulfilled only for a small number of them (depending on the thickness of the sample). However, for off-Bragg angles of the readout beam, only those parasitic grating vectors that lie on the intersection of the Ewald sphere of the readout wave with the primary and conjugate spheres of the recorded PGs result in scattered waves. Geometrically, those intersections are cones and therefore are visible as sharp circles on the flat screen. The apex angles of these cones depend on the readout angle and the ratio of the pump and readout wavelengths [39]. An example for these bright, sharp circles on top of the broad scattering pattern is shown in Figure 7f. Off-Bragg readout clearly reduces light-induced scattering but, of course, also the diffraction efficiency and, thus, the signal of the elementary grating (see Figure 6).

### 5.4. Conclusions

We performed a detailed investigation of holographic gratings recorded in PEGDMA:IL composites for two different recording times and a series of thicknesses by analyzing the diffraction pattern, conducting 2BC experiments, as well as measurements of the angular dependence of the diffraction efficiencies $E_{\pm 2,1,0}$. To evaluate the relevant physical parameters from the latter experiments, we employ a five-wave coupling theory for mixed refractive-index and extinction gratings accounting for a relative phase shift. Moreover, the model also includes a decay of the gratings along the thickness. For the latter reason, we could solve the coupled wave equations only numerically and fitted this to the data keeping $n_{1},\alpha_{1},\varphi ,n_{2},d,L$ as free parameters. We can summarize the findings as follows:

- We found that PEGDMA:IL composites are interesting materials for recording holographic patterns. We observed gratings of the mixed type, refractive-index and extinction gratings. Up to a thickness of about $d_{0}\approx 85\;\mu m$, the first is dominating, whereas for larger thicknesses, extinction becomes increasingly important. The extinction originates from recording PGs simultaneously with the desired elementary grating [76].
- When we focus on the applicability of these gratings, we have to search for a tradeoff between making samples thicker to increase the diffraction efficiency and paying at the same time with a reduction of the grating quality, due to a decay of the pattern strength along the sample thickness and/or enhanced parasitic scattering. Therefore, gratings with up to $d_{0}\approx 85\;\mu m$ are recommended.
- For the thicker samples, a non-sinusoidal shape of the grating with negative second order Fourier coefficients becomes evident. It is interesting to note that in such cases, due to multi-wave coupling, the phase problem of scattering is resolved.
- We anticipate that PEGDMA-IL gratings with a smaller grating spacing are interesting candidates for cold neutron diffractive optical elements. We intend to use these gratings for cold and very cold neutrons [23,80,81,82], in particular looking forward to finally adapt the samples with magnetic liquids (ferro fluids) [83] that couple to the neutron spin. A further batch of IL-polymer composite gratings with a spacing of about half a micron is currently being analyzed.

Thus, IL-polymer composites turn out to be promising materials for holographic applications with high diffraction efficiencies at moderate thicknesses. Then, even the unavoidable detrimental scattering from PGs is still negligible. Further studies of these excellent properties for smaller grating spacings and for use in neutron optics are under way.

## Figures and Tables

**Table 1:** Fitting parameters obtained from 2BC data at $\lambda_{r}=355\;\mathrm{nm}$ and $t_{p}=12\;s$ for thin samples. The thickness was taken from the fit to the angular dependence of the diffraction efficiency (see Section 4.5).

$d_{0}\;\left(\mu m\right)$	${\bar{n}}_{1}\;\left(10^{-3}\right)$	${\bar{\alpha }}_{1}\;\left(10^{-3}/\mu m\right)$	φ(rad)
20	(1.95±0.03)	(1.1±0.4)	(2.9±0.4)
50	(1.28±0.02)	(0.7±0.1)	(3.6±0.2)

**Table 2:** Parameters obtained by fitting the angular dependence of the diffraction efficiency for various thicknesses.

$d_{0}$ (μm)→	20	50	85	100	125	150
${\bar{n}}_{1}\;\left(10^{-3}\right)$	2.42 ± 0.09	1.10 ± 0.03	1.19 ± 0.02	2.29 ± 0.02	1.99 ± 0.09	2.18 ± 0.07
${\bar{n}}_{2}\;\left(10^{-4}\right)$	0.9 ± 0.4	0.8 ± 0.4	0.7 ± 0.3	2.4 ± 0.1	−1.8±0.1	−2.6±0.1
${\bar{\alpha }}_{1}\;\left(10^{-4}/\mu m\right)$	1.8 ± 0.7	2.9 ± 0.3	0.8 ± 0.5	4.0 ± 0.4	3.3 ± 0.4	2.2 ± 0.4
d(μm)	14.3 ± 0.1	51.8 ± 0.4	75.1 ± 0.7	89.8 ± 0.3	96.5 ± 0.3	107.1 ± 0.4
d/L	0.19 ± 0.05	0.89 ± 0.05	2.02 ± 0.05	1.58 ± 0.02	0.1 ± 0.1	0.06 ± 0.05
φ(rad)	−0.10 ± 0.06	3.34 ± 0.06	0.7 ± 0.5	2.98 ± 0.07	0.09 ± 0.08	0.0 ± 0.1
$\exp\left(-2\alpha_{0}d\right)$		0.98	0.90	0.74	0.66	0.83
*Q*	1.22 ± 0.02	3.93 ± 0.07	6.0 ± 0.1	6.9 ± 0.1	8.0 ± 0.1	8.3 ± 0.1
$\chi^{2}\;\left(10^{-5}\right)$	0.01	0.7	2.3	17.7	6.5	8.4

## Supplementary Materials

The following are available online at www.mdpi.com/1996-1944/10/1/9/s1.

## Abbreviations

The following abbreviations are used in this manuscript:

2BC	two-beam coupling
PEGDMA	poly(ethylene glycol) dimethacrylate
ILs	ionic liquids
HS	holographic scattering
PGs	parasitic gratings
POM	polarizing optical microscope
