# Supplementary material for: A Comprehensive Study of Photorefractive Properties in Poly(ethylene glycol) Dimethacrylate— Ionic Liquid Composites
Source: Materials (Basel). 2016 Dec 24;10(1):9. doi: 10.3390/ma10010009 (PMC5344600; doi:10.3390/ma10010009)
Supplement: Supplementary file 1 [file materials-10-00009-s001.pdf]

Supplementary Materials: A Comprehensive Study of Photorefractive Properties in Poly(ethylene glycol) Dimethacrylate—Ionic Liquid Composites

Mostafa A. Ellabban, Gašper Glavan, Jürgen Klepp and Martin Fally

1. Morphology of the Gratings Studied by Light Optical Microscopy (Related to Figure 2 of the Paper)

To characterize the morphology of the gratings, we used optical microscopy. Due to the large grating spacing of about 6 μm, this is the tool of choice. Below are pictures taken with a polarizing light optical microscope (Zeiss Axiophot) for gratings of various thicknesses.

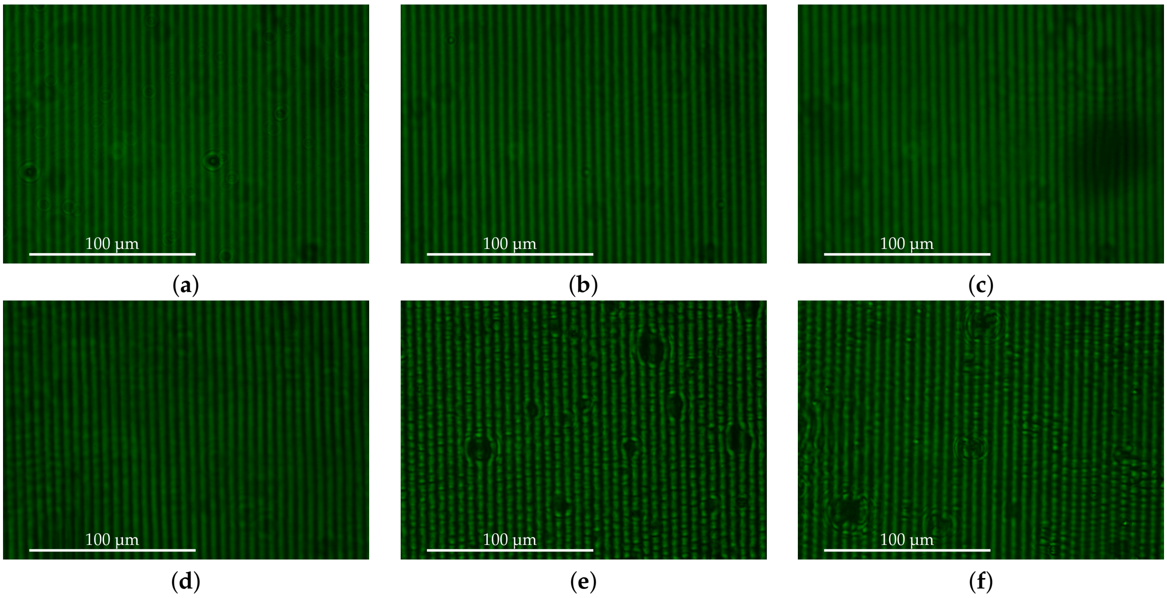

Figure S1. Light optical microscopic pictures ($\lambda_r = 546$ nm) of gratings for various thicknesses ($t_p = 12$ s). (**a**–**f**): $d_0 = 20, 50, 85, 100, 125, 150$ μm.

It can be seen that the morphology is determined by the light-induced phase-separation of the polymeric matrix and the ionic liquid component. The gratings formed are of excellent periodicity. With increasing thickness, droplet forming is more visible, in particular for $d_0 \geq 125$ μm.

2. Temporal Evolution of the Relative Diffraction Efficiency (Related to Figure 4 of the Paper)

The temporal evolution of the relative diffraction efficiency for different thicknesses of the gratings ($t_p = 12$ s) is non-monotonous, as discussed in the paper, and is shown in the following figure.

Note that the use of η is somewhat inappropriate for $d_0 > 100$ μm, as discussed in the paper.

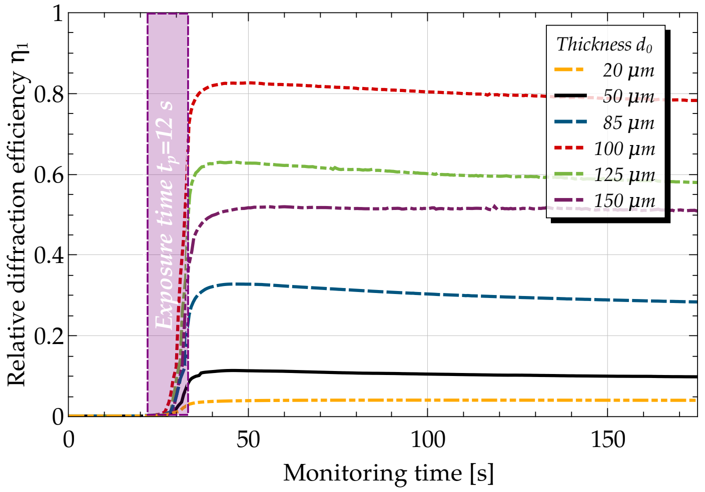

Figure S2. +1st order relative diffraction efficiencies at the Bragg angle as a function of time for gratings of various thicknesses and a recording exposure time of $t_p = 12$ s. The readout wavelength is $\lambda_r = 633$ nm.

3. Angular Dependence of the Diffraction Efficiency (Related to Figure 6 of the Paper)

3.1. Angular Dependence of the Relative Diffraction Efficiency η

As an addendum to Figure 6 of the manuscript, we show the relative diffraction efficiency η_s for all of the samples where this definition is appropriate, i.e., $d_0 \leq 100$ μm (Figure S3).

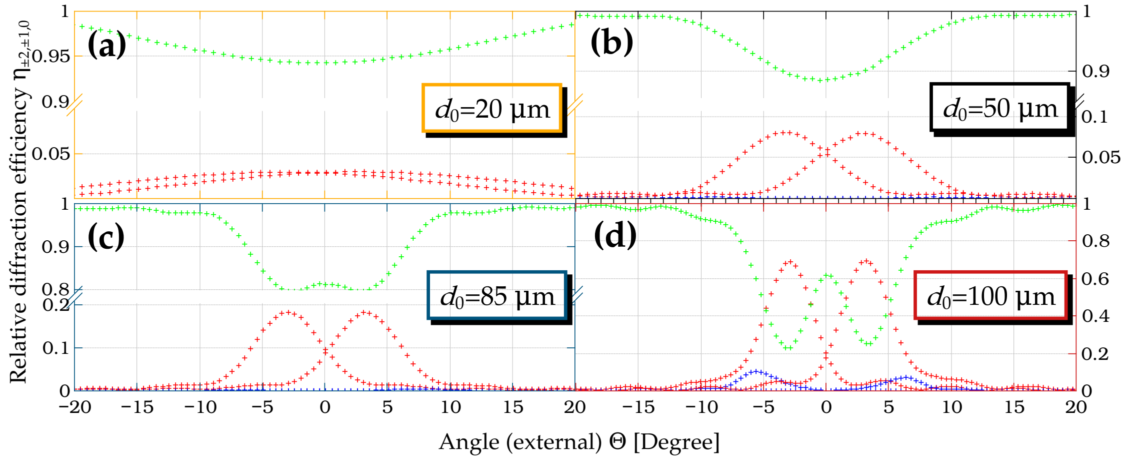

Figure S3. Relative diffraction efficiency for: (**a**) $d_0 = 20$ μm; (**b**) $d_0 = 50$ μm; (**c**) $d_0 = 85$ μm; (**d**) $d_0 = 100$ μm, respectively. $\eta_{\pm 1}$, $\eta_{\pm 2}$, η_0 are indicated by red, blue and green markers, respectively.

To give the reader an idea of the second order contribution also for $d_0 \leq 100$ μm, we add logarithmic plots (Figure S4).

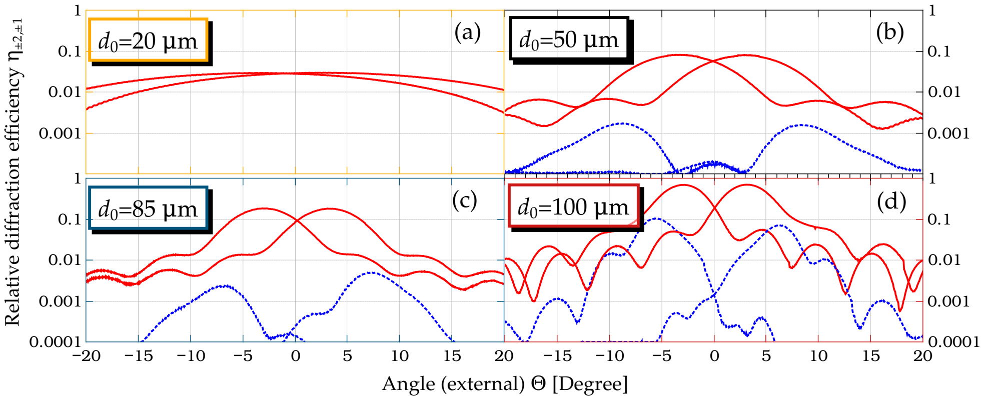

Figure S4. Relative diffraction efficiency in the logarithmic scale for: (**a**) $d_0 = 20$ µm; (**b**) $d_0 = 50$ µm; (**c**) $d_0 = 85$ µm; (**d**) $d_0 = 100$ µm, respectively. $\eta_{\pm 1}$ and $\eta_{\pm 2}$ are indicated by red and blue lines; η_0 is not shown.

3.2. Angular Dependence of the Diffraction Efficiency E

Figure S5 shows the diffraction efficiency E_s for all investigated thicknesses.

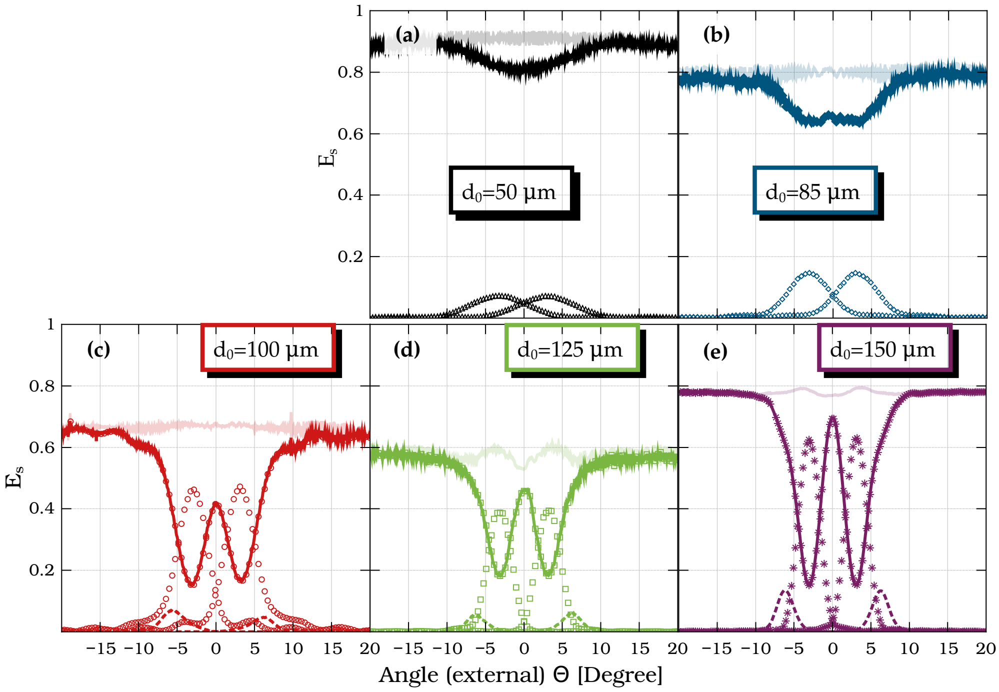

Figure S5. Diffraction efficiency E_s for: (**a**) $d_0 = 50$ µm; (**b**) $d_0 = 85$ µm; (**c**) $d_0 = 100$ µm; (**d**) $d_0 = 125$ µm; (**e**) $d_0 = 150$ µm, respectively. Zero, first and second diffraction orders, as well as the sum of all diffraction orders are indicated by filled markers, open markers, dotted lines and faint lines, respectively (as in Figure 6).

4. Diffraction Regime and Klein–Cooke Parameter (Related to Section 4.5 of the Paper, Figures 6 and 9)

It has been pointed out in a series of papers by Gaylord, Magnusson and Moharam [1–3] that the use of the popular Klein–Cooke parameter Q is not appropriate in certain cases to discriminate between thick and thin gratings. The problem can be understood by the fact that Q is independent of the refractive-index modulation n_1, which, of course, decisively limits the use of Q.

In their papers, Gaylord et al. identified three diffraction regimes: the Bragg regime (only two waves with considerable amplitudes propagate in the grating provided that the Bragg condition is fulfilled) [1], the Raman–Nath regime (a large number of waves propagates at the same time) [2] and the intermediate regime. For the latter, the rigorous coupled wave analysis (RCWA) has to be applied to correctly describe the diffraction properties. To discriminate between these cases, the following inequalities have to be considered [3]:

$$Q'\nu > 1 \quad \wedge \quad Q'/\nu > 20 \qquad \text{Bragg regime} \tag{S1}$$

$$Q'\nu < 1 \quad \wedge \quad Q'/\nu < 20 \qquad \text{Raman-Nath regime,} \tag{S2}$$

where:

$$Q' = \frac{2\pi\lambda d}{n_0'\Lambda^2\cos\theta} \tag{S3}$$

$$\nu = n_1'\pi d/(\lambda\cos\theta), \tag{S4}$$

and θ is the angle of incidence in the medium. In the case that one of the inequalities is obeyed, but the other is violated, the use of the RCWA is mandatory (intermediate regime).

Now, let us have a look at Table 2 of the manuscript: we find that $1 < Q < 10$ for all of our samples, and thus, they meet the criteria for the intermediate regime. By inspecting Figure 6, it is obvious that for $d_0 = 20$ µm, the diffraction is similar to what is expected for a "thin" grating, whereas this not the case for, e.g., the grating with thickness $d_0 > 125$ µm (similar to a "thick" grating).

Figure S6 shows the diffraction regimes according to [3]. The figure is interpreted as follows: the black lines divide the parameter space $(log_{10}(\nu), log_{10}(Q'))$ into four regions:

- Bragg diffraction regime ("thick grating"): only two diffraction orders exist, and their relative diffraction efficiencies are $\eta_0 = \cos^2(\nu), \eta_1 = \sin^2(\nu)$ within a certain error, say 1%.
- Raman–Nath regime ("thin grating"): a vast number of diffraction orders exist simultaneously, and their relative diffraction efficiencies are given by $\eta_s = J_s^2(2\nu)$ for $s = 0, \pm 1$; here, J_s is the s-th order ordinary Bessel function of the first kind.
- RCWA regime: this occurs if just one of the inequalities is fulfilled. It is the inconvenient case, in which the grating is neither "thick" nor "thin".

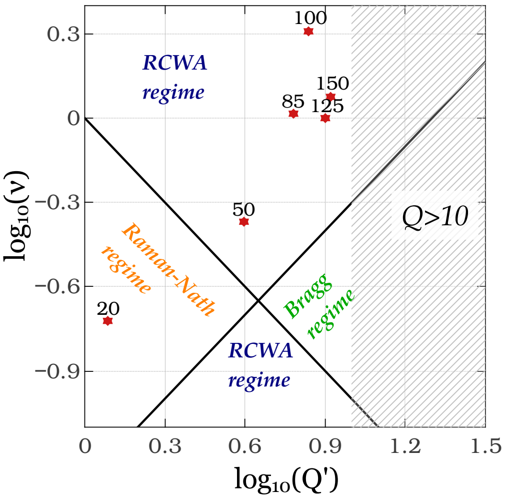

Figure S6. Discrimination between different diffraction regimes: black lines divide the parameter space $(log_{10}(\nu), log_{10}(Q'))$ into four regions: Raman-Nath regime, Bragg regime and RCWA regime (two-fold). The labeled symbols visualize that except for $d_0 = 20$ µm, the gratings are neither thin nor thick. The hatched region indicates where the Klein–Cooke criterion for thick gratings is met ($Q > 10$).

Finally, we would like to mention that the discussion given here neglects any contribution originating from extinction gratings.

References

1. Moharam, M.G.; Gaylord, T.K.; Magnusson, R. Criteria for Bragg Regime Diffraction by Phase Gratings. *Opt. Commun.* **1980**, *32*, 14–18.
2. Moharam, M.G.; Gaylord, T.K.; Magnusson, R. Criteria for Raman-Nath Regime Diffraction by Phase Gratings. *Opt. Commun.* **1980**, *32*, 19–23.
3. Gaylord, T.K.; Moharam, M.G. Thin and thick gratings: Terminology clarification. *Appl. Opt.* **1981**, *20*, 3271–3273.
